# Mechanical Occlusion Chemically Assisted Ablation (MOCA) for Saphenous Vein Insufficiency: A Meta-Analysis of a Randomized Trial

**DOI:** 10.1155/2020/8758905

**Published:** 2020-01-29

**Authors:** Johanes Nugroho, Ardyan Wardhana, Cornelia Ghea

**Affiliations:** ^1^Department of Cardiology and Vascular Medicine, Faculty of Medicine, Universitas Airlangga, Surabaya, Indonesia; ^2^Dr. Soetomo General Hospital, Surabaya, Indonesia; ^3^PILAR Research and Education, Cambridge, UK

## Abstract

**Purpose:**

A previous meta-analysis has conducted nonrandomized trials for mechanochemical ablation (MOCA). Since medium-term follow-up data from randomized clinical trials (RCTs) are becoming available, we chose to perform a meta-analysis of RCTs to assess the efficacy and safety of MOCA for saphenous vein insufficiency.

**Methods:**

A systematic search of all RCTs comparing the anatomical success of MOCA for saphenous vein insufficiency to thermal ablation was performed using the PubMed and Cochrane Library databases. We employed the Mantel-Haenszel random-effects meta-analysis of outcomes using RevMan 5.3.

**Results:**

Four studies (615 patients) were included in this meta-analysis. The MOCA group had 93.4% and 84.5%, whereas the thermal ablation group had 95.8% and 94.8% of anatomical success rate at 1 month (short-term) and a period of more than 6 months but less than 1-year follow-up (mid-term), respectively. According to intention-to-treat analysis, there were similar anatomical successes in MOCA and thermal ablation groups at the short-term follow-up (low-quality evidence; relative risk (RR) = 0.98 (95% CI, 0.94–1.03); *P* = 0.44; *I*^2^ = 53%). The estimated effect of MOCA on anatomical success showed a statistically significant reduction at the mid-term follow-up (moderate-quality evidence; RR = 0.89 (95% CI, 0.84–0.95); *P* = 0.0002; *I*^2^ = 0%). MOCA had fewer incidence of nerve injury, deep vein thrombosis, and skin burns compared to the thermal ablation procedure (low-quality evidence; RR = 0.33 (95% CI, 0.09–1.28); *P* = 0.11; *I*^2^ = 0%).

**Conclusion:**

MOCA offered fewer major complications but lesser anatomical success at the period of more than 6 months but less than 1-year follow-up than thermal ablation. *Trial Registration*. This trial is registered with UMIN Clinical Trial Registry (UMIN ID 000036727).

## 1. Introduction

Saphenous vein insufficiency is the most common chronic venous incompetence of lower limbs. It affects more than 30% of adults [1]. The first-line treatment of venous incompetence is thermal ablation, e.g., radiofrequency ablation (RFA) and endovenous laser ablation (EVLA) [2]. However, the use of heat energy in thermal ablation was associated with complications such as nerve injury and skin burns [3, 4].

To avoid thermal energy complications, few novel treatments such as sclerotherapy, cyanoacrylate, and mechanochemical ablation (MOCA) have been introduced. One of MOCA device is ClariVein (Merit Medical, South Jordan, Utah) that combines mechanical injury to the venous endothelium using a rotating wire with simultaneous delivery and dispersion of a liquid sclerosant [5]. The liquid sclerosant causes irreversible damage to the cellular membranes of the endothelium, resulting in the fibrosis of veins [6]. Another technique to produce a nontumescent sclerosant-assisted ablation is by means of Flebogrif (Balton, Poland), a device that is scratching the endothelium lining by a dedicated catheter.

A previous meta-analysis has conducted nonrandomized trials for MOCA [7]. It is important to study the use of MOCA since medium-term follow-up data on randomized clinical trials are now becoming available. Therefore, we performed a meta-analysis of randomized trials aiming to assess the safety and efficacy of MOCA by comparing thermal ablation for saphenous vein insufficiency.

## 2. Methods

All RCTs comparing the anatomical success of MOCA for saphenous vein insufficiency (great saphenous vein (GSV), small saphenous vein (SSV), or both) to the success rate of a thermal ablation procedure were eligible for inclusion in this review. All trials comparing MOCA to treatments other than thermal ablation were excluded. A systematic search of the literature was undertaken on July 7, 2019, after receiving an approval from the Institutional Review Board. Two different databases (PubMed and Cochrane Library) were used to perform a systematic search of all the literature without language restriction. Search terms were differently spelled text words or abbreviations, such as “varicose,” “saphenous incompetence,” “saphenous insufficiency,” “saphenous varicose veins,” “saphenous reflux,” “saphenous vein,” “endovenous ablation,” “clarivein,” “MOCA,” “mechanochemical ablation,” “randomized,” and “rct” in the title, abstract, and medical subject heading (MeSH). Reference lists of the included studies were also evaluated to identify additional relevant studies. We followed the Preferred Reporting Items for Systematic Reviews and Meta-Analyses guidelines for research reporting.

Two investigators independently screened and assessed titles and abstracts before full-text retrieval. The full papers that potentially met the inclusion/exclusion criteria were reviewed by the two authors for final inclusion. Subsequently, two investigators extracted the data, which included authors, year of publication, detailed intervention agent, veins treated (either GSVs or SSVs), postprocedural management, number of patients, primary outcomes, and adverse events. The corresponding author was contacted to obtain any incomplete data. All extracted data were recorded with a dedicated data extraction form on an Excel spreadsheet.

The primary outcome in our meta-analysis was anatomical success that was defined either as complete occlusion of the saphenous vein or at least proximal occlusion (>5 cm proximally occluded with >5 cm distally open) on duplex ultrasound (DUS) imaging. The primary outcome was categorized at the short-term follow-up (1 month) and mid-term follow-up (≥6 months but ≤1 year). Secondary outcomes were phlebitis and major adverse event rates, including nerve injury, DVT, and skin burns. Nerve injury was reported differentially in the studies as sensory disturbance, paresthesia, or numbness. Other minor complications (e.g., hematoma, lumps, and hyperpigmentation) were excluded from the analysis.

Two authors independently assessed the methodological quality of the articles using the Risk of Bias Tool (Cochrane collaboration). They employed a GRADE approach to evaluate the quality of evidence [8, 9]. Factors assessed for the risk of bias were as follows [1]: randomization [2], adherence to intervention [3], measurement [4], missing outcome data, and reporting [5]. We evaluated the GRADE while taking into account the following factors: risk of bias, imprecision, indirectness of evidence, inconsistency in results among included studies, and reporting bias.

We conducted the meta-analysis using relative risk (RR) for anatomical success rates at short-term and mid-term follow-up periods. We employed the Mantel−Haenszel random-effects method using Review Manager (RevMan v5.3 2014). We conducted an intention-to-treat (ITT) analysis using imputation data according to event rates among completers in the separate groups [10]. Available case analysis was further performed as a sensitivity analysis. We evaluated between and within-study heterogeneity using the *I*^2^ statistic.

## 3. Results

**Figure 1:**
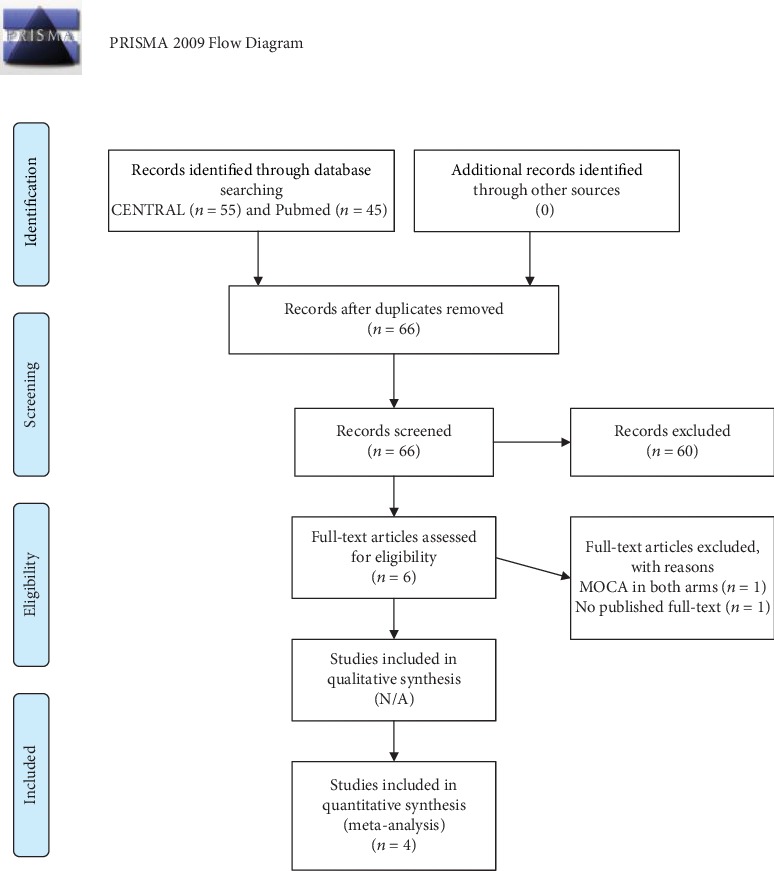
PRISMA flow diagram.

A total of 100 studies were identified after our initial search was completed (45 citations in PubMed and 55 in Cochrane Library, as presented in Figure 1). Six studies were further retrieved to review the full text. However, two studies were excluded because one lacked a control and one was an in-abstract study [11, 12]. Therefore, four studies were finally included in our analysis [13–16]. Attempts to contact authors to acquire unpublished data were uniformly unsuccessful. As such, only information available from the publication was used.  Table 1 summarizes the clinical characteristics of the included studies.

**Figure 2:**
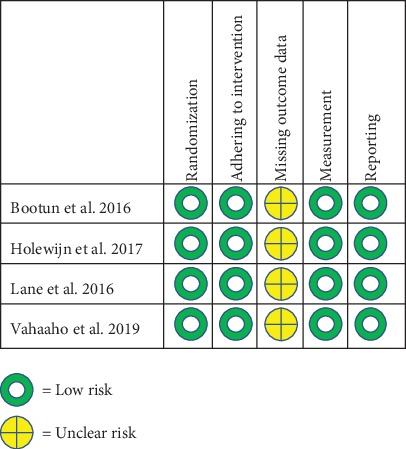
Risk of bias assessment.

The Cochrane risk of bias analysis is shown in Figure 2. There was a low risk of a randomization process in all included trials. Blinding to participants and the delivering clinicians was not performed in all included trials. There was no placebo included for comparison. However, there was assessor-blinding when evaluating the anatomical success using DUS imaging. One trial was stopped early because reimbursement of MOCA was suspended [13]. Participants who were not followed up ranged from 6% to 34%, and there were no documented reasons for loss to follow-up. All trials reported the results from available case analyses and not ITT analysis.

**Figure 3:**
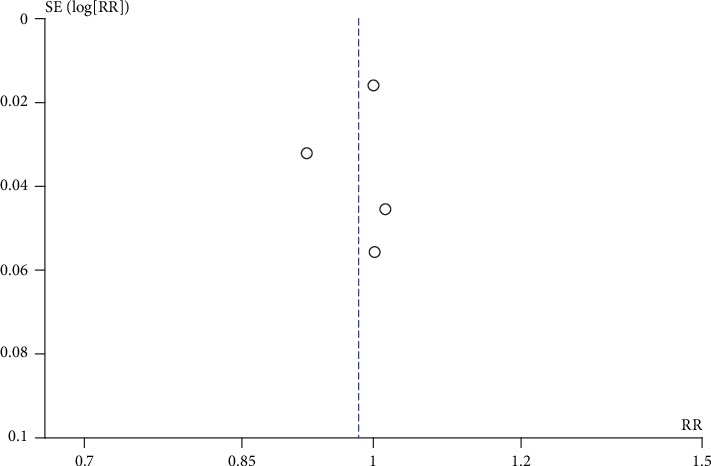
Funnel plot of comparisons: mechanochemical ablation vs thermal ablation intention-to-treat analysis, outcome: anatomical success short-term.

Table 2 shows the quality of evidence, and the GRADE approach was used for each outcome. We did not downgrade from the risk of bias aspect because most information was from studies that were at low risk of bias. We downgraded one level for the evidence of anatomical success at the short-term follow-up because there was substantial heterogeneity. Indirectness did not appear to be an issue in all outcomes. We downgraded the evidence of major complications for the imprecision aspect because there was a wide CI that included no important effect and RR reduction greater than 25%. The publication bias was assessed for all outcomes because there was asymmetry from a visual inspection of the funnel plot (Figure 3). This finding was not further evaluated by conducting Egger's test because there were less than 10 included trials.

The MOCA group had 93.4% of anatomical success rate, whereas the thermal ablation group had 95.8% at the 1-month follow-up. According to the meta-analysis using ITT as shown in Table 3, there were similar anatomical successes in the MOCA and thermal ablation groups at the 1-month follow-up (4 trials; RR = 0.98 (95% CI, 0.94–1.03); *P* = 0.44); however, there was substantial heterogeneity (*I*^2^ = 53%; *P* = 0.09). At the mid-term follow-up (≥6 months but ≤1 year), the MOCA group had 84.5% of anatomical success rate, whereas the thermal ablation group had 94.8% (Table 4). The estimated effect of MOCA on anatomical success showed a statistically significant reduction at the mid-term follow-up (3 trials; RR = 0.89 (95% CI, 0.84–0.95); *P* = 0.0002), without substantial heterogeneity (*I*^2^ = 0%; *P* = 0.52).

We performed sensitivity analysis using the available case analyses. The direction of effect did not change for anatomical success rates. MOCA did not decrease anatomical success compared to thermal ablation at the 1-month follow-up (4 trials; RR = 0.98 (95% CI, 0.93–1.03); *P* = 0.46); however, there was substantial heterogeneity (*I*^2^ = 56%; *P* = 0.08). The RR for anatomical success at the mid-term follow-up (≥6 months but ≤1 year) was significantly lower after treatment with MOCA than that with thermal ablation (3 trials; RR = 0.89 (95% CI, 0.84–0.96); *P* = 0.001), without substantial heterogeneity (*I*^2^ = 0%; *P* = 0.67).

Table 5 shows the details of adverse events in the four included trials. MOCA had fewer major complications compared to thermal ablation procedures (4 trials, RR = 0.33 (95% CI, 0.09–1.28); *P* = 0.11; *I*^2^ = 0%). However, participants in the MOCA group had a higher risk of phlebitis than the thermal ablation group (4 trials, RR = 1.39 (95% CI, 0.67–2.86); *P* = 0.37; *I*^2^ = 0%).

## 4. Discussion

We report that MOCA had a similar anatomical success rate with low-quality evidence at the 1-month follow-up, but there was a reduction of the anatomical success rate with moderate-quality evidence at the period of more than 6 months but less than 1-year follow-up compared with thermal ablation. Previous meta-analyses that included nonrandomized trials demonstrated that MOCA could be considered an effective treatment for saphenous vein insufficiency; however, these results were based on a pooled analysis of low-quality data [17]. In previous studies, the pooled anatomical success rate at the mid-term follow-up was 91%, 97.1%, and 98.5% for MOCA, RFA, and EVLA, respectively [7, 18]; the rates obtained in our study were lower: 84.5% for MOCA and 94.8% for thermal ablation. Blinding to the assessor may have contributed to the lower anatomical success rate observed in our study.

The major benefit of MOCA reported in previous reviews was pain intensity reduction during the procedure [17, 18]. However, we did not perform an analysis of pain intensity because there was a high risk of measurement bias from no participant blinding in the available trials. The potential benefits in reducing the risk of nerve injury might be of considerable clinical importance for MOCA. Our meta-analysis indicated that MOCA had a lower risk of major complications compared with thermal ablation. Nerve injury was seen in 0.4% and 3.2% cases after MOCA and thermal ablation, respectively.

There was disparity in terms of anatomical success measure definitions, postprocedure management, and technical aspects of the MOCA procedure. Holejwin et al. reported different results from the others as they used a different sclerosant and categorized DUS image findings into 3 categories instead of 4 [13]. The types of sclerosant used by different studies may have influenced anatomical success. There was substantial heterogeneity in anatomical success at the short-term follow-up in our study. Removing those trials improved the heterogeneity without changing the direction of the effect. Therefore, the heterogeneity in our study might be due to various types of sclerosants and definitions of anatomical success.

There were a few limitations in our meta-analysis. First, we evaluated efficacy only from the anatomical success rate aspect, not from pain reduction, clinical success, or quality of life aspects. Second, there was a wide CI around the effect estimate of adverse event outcomes because there were few events in this meta-analysis. Future trials are required. To date, there is one unpublished trial and one ongoing trial investigating the anatomical success of MOCA compared with thermal ablation [12, 19]. Lastly, the need of data at more than 1 year of outcome measurement at least is suggested.

## 5. Conclusion

Pooled analysis from limited trials demonstrated that MOCA offered fewer major complications (DVT, nerve injury, and skin burns) but lesser anatomical success at the period of more than 6 months but less than 1-year follow-up than thermal ablation. More RCTs together with proper homogeneous data collection are needed to reach optimal information size.

## Figures and Tables

**Table 1:** The characteristics of the included trials.

Author	Vein insufficiency	Intervention	Control	Postprocedural management	DUS scan finding	Follow-up period (percentage lost-to follow-up in groups MOCA and thermal ablation)	Adverse event identification
Holewijn et al. 2017 [13]	GSV	MOCA using ClariVein and polidocanol 3% 2 ml for the first 10-15 cm and 1.5% for the remainder. Pullback rate 7 s/cm	RFA using Closurefast	Phlebotomy as indicated, stocking compression for 2 weeks	Complete occlusion, partial recanalization >10 cm, complete recanalization	1 month (0% & 0%), 12 months (21.4% & 30.1%), 24 months (26.2% & 21.4%)	Major: DVT, pulmonary embolism, skin burn, saphenous neuralgia Minor: phlebitis, induration, hematoma, hyperpigmentation

Vähäaho et al. 2019 [14]	GSV	MOCA using ClariVein and STS 1.5%. Pullback rate was not defined	RFA using Closurefast or EVLA using ELVes 1470-nm diode radial laser	Phlebotomy for every patient. Other postprocedural management was not defined	Complete occlusion, proximal occlusion, distal occlusion, complete recanalization	1 month (0% & 0%), 12 months (18.2% & 6.1%)	Major: DVT, nerve injury Minor: hyperpigmentation, hematoma, phlebitis, lumps

Bootun et al. 2016 [15]	GSV, SSV	MOCA using ClariVein and STS 2%. Pullback rate was not defined	RFA using Venefit	Phlebotomy as indicated, stocking compression for 2 weeks	Complete occlusion, proximal occlusion, distal occlusion, complete recanalization	1 month (13.3% & 55.9%)	Major: DVT Minor: phlebitis

Lane et al. 2016 [16]	GSV, SSV	MOCA using ClariVein and STS 2%. Pullback rate 7 s/cm	RFA using Venefit	Phlebotomy as indicated, LMWH administration, stocking compression for 2 weeks	Complete occlusion, proximal occlusion, distal occlusion, complete recanalization	1 month (16.9% & 26.8%), 6-months (25.3% & 28%)	Major: DVT, sensory disturbance Minor: phlebitis, hyperpigmentation

**Table 2:** GRADE assessment.

Comparison	Outcomes	Risk of bias	Inconsistency	Indirectness	Imprecision	Publication of bias	GRADE
Anatomical success	Short-term	No serious	Some	No at all	No	Presence	Low
	Mid-term	No serious	No	No at all	No	Presence	Moderate
Complication	Major complication	No serious	No	No at all	Some	Presence	Low
	Phlebitis	No serious	No	No at all	No	Presence	Moderate

**Table 3:** Summary of findings.

		Studies	Participants	Effect estimate	Heterogeneity
ITT analysis	Anatomical success at short-term	4	615	RR 0.98 [0.94, 1.03]; *P* = 0.44	*P* = 0.09; *I*^2^ = 53%
	Anatomical success at mid-term	3	496	RR 0.89 [0.84, 0.95]; *P* = 0.0002	*P* = 0.53; *I*^2^ = 0%
Available case analysis	Anatomical success at short-term	4	532	RR 0.98 [0.93, 1.03]; *P* = 0.46	*P* = 0.08; *I*^2^ = 56%
	Anatomical success at mid-term	3	386	RR 0.89 [0.84, 0.96]; *P* = 0.001	*P* = 0.67; *I*^2^ = 0%
Adverse events	Major complication	4	615	RR 0.33 [0.09, 1.28]; *P* = 0.11	*P* = 0.73; *I*^2^ = 0%
	Phlebitis	4	615	RR 1.39 [0.67, 2.86]; *P* = 0.37	*P* = 0.58; *I*^2^ = 0%

**Table 4:** Anatomical success and complication rates in each study.

	Anatomical success rates		DVT		Sensory disturbances	
Studies	MOCA	Thermal ablation	MOCA	Thermal ablation	MOCA	Thermal ablation
	Short-term		At the end of study		At the end of study	
Bootun et al. 2016 [15]	91.7%	91.5%	0%	1.7%	0%	0%
Holewijn et al. 2017 [13]	91.3%	99.0%	0%	0%	1.0%	2.9%
Lane et al. 2016 [16]	92.8%	91.5%	1.2%	1.2%	0%	0%
Vähäaho et al. 2019 [14]	100.0%	100.0%	0%	0%	0%	7.6%
Total	93.4%	95.8%	0.7%	1.4%	0.4%	3.2%
	Mid-term					
Lane et al. 2016 [16]	86.7%	92.7%				
Vähäaho et al. 2019 [14]	86.4%	100.0%				
Holewijn et al. 2017 [13]	81.6%	93.2%				
Total	84.5%	94.8%				

**Table 5:** Adverse events in the included trials.

Adverse events	Phlebitis		DVT		Nerve injury	
	MOCA	Thermal ablation	MOCA	Thermal ablation	MOCA	Thermal ablation
Holewijn et al. 2017 [13]	12	8	0	0	1	3
Vähäaho et al. 2019 [14]	1	0	0	0	0	5
Bootun et al. 2016 [15]	0	2	0	1	Not reported	Not reported
Lane et al. 2016 [16]	3	2	1	1	0	0

## Data Availability

The data supporting this meta-analysis are from previously reported studies and datasets, which have been cited. The processed data are available in the supplementary files (available here).

## Supplementary Materials

Supplementary Materials Forest plot of all outcomes in meta-analysis.
