# Supplementary material for: Mechanical Occlusion Chemically Assisted Ablation (MOCA) for Saphenous Vein Insufficiency: A Meta-Analysis of a Randomized Trial
Source: Int J Vasc Med. 2020 Jan 29;2020:8758905. doi: 10.1155/2020/8758905 (PMC7204279; doi:10.1155/2020/8758905)
Supplement: Supplementary Materials — Forest plot of all outcomes in meta-analysis. [file 8758905.f1.pdf]

1 MOCA vs thermal ablation ITT analysis

1.1 Anatomical success mid-term

	MOCA		Thermal ablation			Risk Ratio		R
Study or Subgroup	Events	Total	Events	Total	Weight	M-H, Random, 95% CI	Year	A
Lane 2016	72	83	76	82	34.0%	0.94 [0.84, 1.04]	2016	+
Vahaaho 2019	51	59	66	66	33.3%	0.86 [0.78, 0.96]	2018	+
Holewijn 2017	84	103	96	103	32.8%	0.88 [0.79, 0.97]	2019	+
**Total (95% CI)**		**245**		**251**	**100.0%**	**0.89 [0.84, 0.95]**		
Total events	207		238					

Heterogeneity: Tau² = 0.00; Chi² = 1.29, df = 2 (P = 0.53); I² = 0%
Test for overall effect: Z = 3.71 (P = 0.0002)

Risk Ratio
M-H, Random, 95% CI

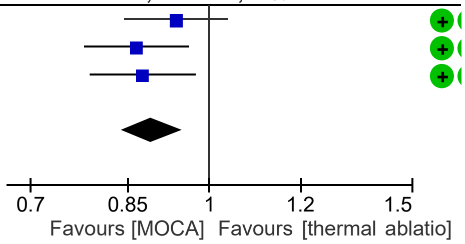

Risk of bias legend
(**A**) Randomization process
(**B**) Adhering to intervention
(**C**) Missing outcome data
(**D**) Measurement
(**E**) Reporting

1.2 Anatomical success short-term

	MOCA		Thermal ablation			Risk Ratio	Risk o		
Study or Subgroup	Events	Total	Events	Total	Weight	M-H, Random, 95% CI	A	B	C
Bootun 2016	55	60	54	59	14.0%	1.00 [0.90, 1.12]	+	+	?
Holewijn 2017	94	103	102	103	26.8%	0.92 [0.87, 0.98]	+	+	?
Lane 2016	77	83	75	82	18.2%	1.01 [0.93, 1.11]	+	+	?
Vahaaho 2019	59	59	66	66	41.0%	1.00 [0.97, 1.03]	+	+	?
**Total (95% CI)**		**305**		**310**	**100.0%**	**0.98 [0.94, 1.03]**			
Total events	285		297						

Heterogeneity: Tau² = 0.00; Chi² = 6.38, df = 3 (P = 0.09); I² = 53%
Test for overall effect: Z = 0.78 (P = 0.44)

Risk Ratio
M-H, Random, 95% CI

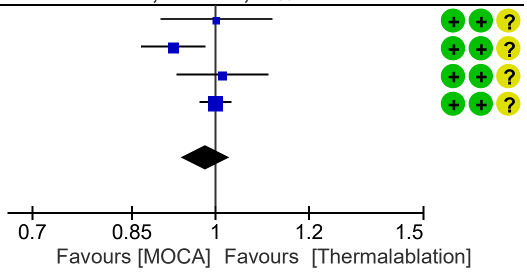

Risk of bias legend
(**A**) Randomization process
(**B**) Adhering to intervention
(**C**) Missing outcome data
(**D**) Measurement
(**E**) Reporting

2 MOCA vs thermal ablation PP analysis

2.1 Anatomical success mid-term

	MOCA		Thermal ablation			Risk Ratio	
**Study or Subgroup**	**Events**	**Total**	**Events**	**Total**	**Weight**	**M-H, Random, 95% CI**	**Year**
Lane 2016	54	62	55	59	31.7%	0.93 [0.83, 1.05]	2016
Vahaaho 2019	48	55	62	62	39.7%	0.87 [0.79, 0.97]	2018
Holewijn 2017	66	81	62	67	28.6%	0.88 [0.78, 1.00]	2019
**Total (95% CI)**		**198**		**188**	**100.0%**	**0.89 [0.84, 0.96]**	
Total events	168		179				

Heterogeneity: Tau² = 0.00; Chi² = 0.79, df = 2 (P = 0.67); I² = 0%
Test for overall effect: Z = 3.30 (P = 0.0010)

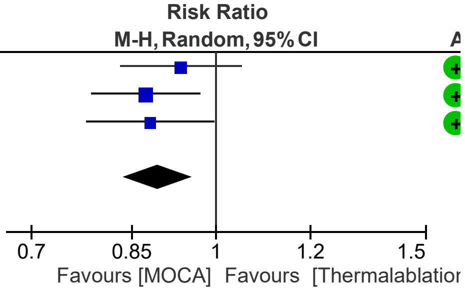

Risk of bias legend
(**A**) Randomization process
(**B**) Adhering to intervention
(**C**) Missing outcome data
(**D**) Measurement
(**E**) Reporting

2.2 Anatomical success short-term

	MOCA		Thermal ablation			Risk Ratio	
**Study or Subgroup**	**Events**	**Total**	**Events**	**Total**	**Weight**	**M-H, Random, 95% CI**	**Year**
Bootun 2016	48	52	24	26	11.9%	1.00 [0.87, 1.15]	2016
Lane 2016	64	69	50	55	16.8%	1.02 [0.92, 1.13]	2016
Vahaaho 2019	59	59	66	66	41.9%	1.00 [0.97, 1.03]	2018
Holewijn 2017	94	103	101	102	29.4%	0.92 [0.87, 0.98]	2019
**Total (95% CI)**		**283**		**249**	**100.0%**	**0.98 [0.93, 1.03]**	
Total events	265		241				

Heterogeneity: Tau² = 0.00; Chi² = 6.76, df = 3 (P = 0.08); I² = 56%
Test for overall effect: Z = 0.75 (P = 0.46)

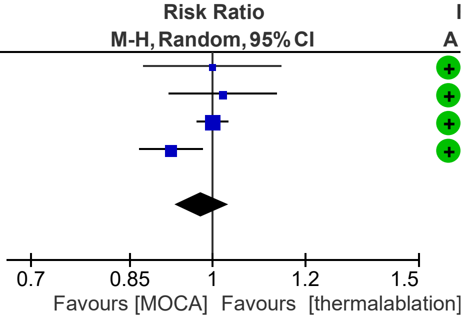

Risk of bias legend
(**A**) Randomization process
(**B**) Adhering to intervention
(**C**) Missing outcome data
(**D**) Measurement
(**E**) Reporting

5 Complication

5.1 Phlebitis

Study or Subgroup	MOCA Events	MOCA Total	Thermal ablation Events	Thermal ablation Total	Weight	Risk Ratio M-H, Random, 95% CI	Risk of Bi A	B	C	D
Bootun 2016	0	60	2	59	5.8%	0.20 [0.01, 4.01]	+	+	?	+
Holewijn 2017	12	103	8	103	72.2%	1.50 [0.64, 3.52]	+	+	?	+
Lane 2016	3	83	2	82	16.9%	1.48 [0.25, 8.64]	+	+	?	+
Vahaaho 2019	1	59	0	66	5.2%	3.35 [0.14, 80.69]	+	+	?	+
**Total (95% CI)**		**305**		**310**	**100.0%**	**1.39 [0.67, 2.86]**				
Total events	16		12							

Heterogeneity: Tau² = 0.00; Chi² = 1.97, df = 3 (P = 0.58); I² = 0%

Test for overall effect: Z = 0.89 (P = 0.37)

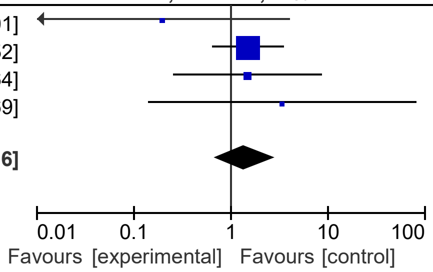

Risk of bias legend
(**A**) Randomization process
(**B**) Adhering to intervention
(**C**) Missing outcome data
(**D**) Measurement
(**E**) Reporting

5.2 Major complication

Study or Subgroup	MOCA Events	MOCA Total	Thermal ablation Events	Thermal ablation Total	Weight	Risk Ratio M-H, Random, 95% CI	Risk of Bi A	B	C	D
Bootun 2016	0	60	1	59	18.0%	0.33 [0.01, 7.89]	+	+	?	+
Holewijn 2017	1	103	3	103	36.0%	0.33 [0.04, 3.15]	+	+	?	+
Lane 2016	1	83	1	82	24.0%	0.99 [0.06, 15.53]	+	+	?	+
Vahaaho 2019	0	59	5	66	22.0%	0.10 [0.01, 1.80]	+	+	?	+
**Total (95% CI)**		**305**		**310**	**100.0%**	**0.33 [0.09, 1.28]**				
Total events	2		10							

Heterogeneity: Tau² = 0.00; Chi² = 1.31, df = 3 (P = 0.73); I² = 0%

Test for overall effect: Z = 1.60 (P = 0.11)

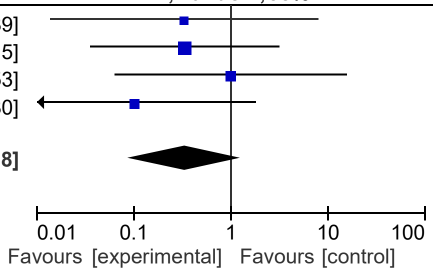

Risk of bias legend
(**A**) Randomization process
(**B**) Adhering to intervention
(**C**) Missing outcome data
(**D**) Measurement
(**E**) Reporting

5.3 DVT

Study or Subgroup	MOCA Events	MOCA Total	Thermal ablation Events	Thermal ablation Total	Weight	Odds Ratio M-H, Random, 95% CI	Risk of Bi A	B	C	D
Bootun 2016	0	60	1	59	42.8%	0.32 [0.01, 8.07]	+	+	?	+
Holewijn 2017	0	103	0	103		Not estimable	+	+	?	+
Lane 2016	1	83	1	82	57.2%	0.99 [0.06, 16.06]	+	+	?	+
Vahaaho 2019	0	59	0	66		Not estimable	+	+	?	+
**Total (95% CI)**		**143**		**141**	**100.0%**	**0.61 [0.07, 5.03]**				
Total events	1		2							

Heterogeneity: Tau² = 0.00; Chi² = 0.27, df = 1 (P = 0.61); I² = 0%
Test for overall effect: Z = 0.46 (P = 0.65)

Odds Ratio M-H, Random, 95% CI

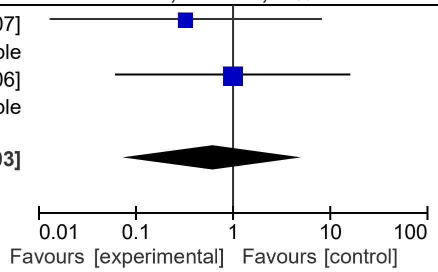

Risk of bias legend
(**A**) Randomization process
(**B**) Adhering to intervention
(**C**) Missing outcome data
(**D**) Measurement
(**E**) Reporting

5.4 Sensoric disturbances

Study or Subgroup	MOCA Events	MOCA Total	Thermal ablation Events	Thermal ablation Total	Weight	Odds Ratio M-H, Random, 95% CI	Risk of Bi A	B	C	D
Bootun 2016	0	0	0	0		Not estimable	+	+	?	+
Holewijn 2017	1	103	3	103	62.1%	0.33 [0.03, 3.19]	+	+	?	+
Lane 2016	0	83	0	82		Not estimable	+	+	?	+
Vahaaho 2019	0	59	5	66	37.9%	0.09 [0.01, 1.74]	+	+	?	+
**Total (95% CI)**		**245**		**251**	**100.0%**	**0.20 [0.03, 1.23]**				
Total events	1		8							

Heterogeneity: Tau² = 0.00; Chi² = 0.46, df = 1 (P = 0.50); I² = 0%
Test for overall effect: Z = 1.74 (P = 0.08)

Odds Ratio M-H, Random, 95% CI

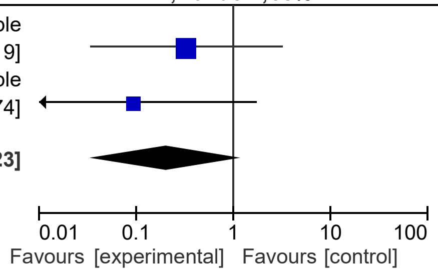

Risk of bias legend
(**A**) Randomization process
(**B**) Adhering to intervention
(**C**) Missing outcome data
(**D**) Measurement
(**E**) Reporting
